# Mass Spectrometry versus Conventional Techniques of Protein Detection: Zika Virus NS3 Protease Activity towards Cellular Proteins

**DOI:** 10.3390/molecules26123732

**Published:** 2021-06-18

**Authors:** Agnieszka Dabrowska, Aleksandra Milewska, Joanna Ner-Kluza, Piotr Suder, Krzysztof Pyrc

**Affiliations:** 1 Virogenetics Laboratory of Virology, Malopolska Centre of Biotechnology, Jagiellonian University, Gronostajowa 7a, 30-387 Krakow, Poland; agnieszka.dabrowska@doctoral.uj.edu.pl (A.D.); aleksandra.milewska@uj.edu.pl (A.M.); 2 Microbiology Department, Faculty of Biochemistry, Biophysics and Biotechnology, Jagiellonian University, Gronostajowa 7a, 30-387 Krakow, Poland; 3 Department of Analytical Chemistry and Biochemistry, Faculty of Materials Science and Ceramics, AGH University of Science and Technology, Mickiewicza 30, 30-059 Krakow, Poland; nerkluza@agh.edu.pl

**Keywords:** Zika virus, translation initiation factor eIF4G1, proteomics, viral NS2B-NS3 protease

## Abstract

Mass spectrometry (MS) used in proteomic approaches is able to detect hundreds of proteins in a single assay. Although undeniable high analytical power of MS, data acquired sometimes lead to confusing results, especially during a search of very selective, unique interactions in complex biological matrices. Here, we would like to show an example of such confusing data, providing an extensive discussion on the observed phenomenon. Our investigations focus on the interaction between the Zika virus NS3 protease, which is essential for virus replication. This enzyme is known for helping to remodel the microenvironment of the infected cells. Several reports show that this protease can process cellular substrates and thereby modify cellular pathways that are important for the virus. Herein, we explored some of the targets of NS3, clearly shown by proteomic techniques, as processed during infection. Unfortunately, we could not confirm the biological relevance of protein targets for viral infections detected by MS. Thus, although mass spectrometry is highly sensitive and useful in many instances, also being able to show directions where cell/virus interaction occurs, we believe that deep recognition of their biological role is essential to receive complete insight into the investigated process.

## 1. Introduction

The Zika virus is a flavivirus discovered in 1947 in primates inhabiting the African Zika forest [1]. Although the virus was found to infect humans, for decades it was not considered to be a medical threat due to limited distribution and very mild symptoms associated with infection. However, more than 10 years ago, interest in the Zika virus began to increase as it became clear that the virus has broadened its geographic distribution, and the first outbreak was reported in the Federated States of Micronesia [2]. In 2015, case definition became more precise, and some data suggested that the infection may be more dangerous than previously thought. While the symptoms are relatively mild and include fever, rash, headache, and muscle pain, the infection may cause severe sequelae, and it is associated with Guillain–Barré syndrome [3]. The infection is most severe in pregnant women since it interferes with the development of the fetal cerebrum, predominantly resulting in microcephaly. A number of in vitro and in vivo studies have confirmed these observations [4,5,6,7,8,9,10].

Flaviviruses are small, enveloped viruses with a positive-strand RNA genome, which is delivered to the target cell as a single-stranded RNA molecule containing a single open reading frame (ORF). This ORF is translated into an immature polyprotein, which is co- and post-translationally cleaved by viral and cellular proteases to yield 10 mature viral proteins: capsid (C), membrane (prM/M), and envelope (E) structural proteins, and seven nonstructural proteins (NS1, NS2A, NS2B, NS3, NS4A, NS4B, and NS5) [11]. Cleavage sites processed by the viral serine NS3 protease are located between NS2A/NS2B, NS2B/NS3, NS3/NS4A, and NS4B/NS5. Furin or similar cellular proteases process the prM/M site, while other host cell proteases reportedly cleave C/prM, prM/E, E/NS1, NS1/NS2A, and NS4A/NS4B sites [12]. The NS3 protein consists of an N-terminal serine protease domain and a C terminal region harboring the RNA helicase, nucleoside triphosphatase, and 5′ RNA triphosphatase activities. Furthermore, the small nonstructural NS2B protein anchors NS3 to the endoplasmic reticulum (ER) membrane [13,14,15,16,17]. The presence or absence of NS2B affects the tertiary structure, activity, and stability of NS3 [18,19,20].

Flaviviral proteases are essential for viral replication; hence, they are considered promising targets for antiviral agents. Indeed, the development of HCV NS3/NS4A protease inhibitors proved a breakthrough in hepatitis C therapy, and these drugs received U.S. Food and Drug Administration (FDA) and European Medicines Agency (EMA) approval for use in humans [21,22,23]. Interestingly, flaviviral proteases were reported to modify the cellular microenvironment significantly. The cleavage of host proteins is beneficial for the virus by diminishing the cellular responses, cellular remodeling metabolism, and other mechanisms. Such a strategy is common for viruses, as exemplified by human rhinovirus (HRV) that modulates apoptosis by cleaving receptor-interacting protein kinase-1 (RIPK1) at the noncanonical site and blocking caspase 8 mediated activation of the pathway [24]. Interestingly, the picornaviral protease processes translation initiation factor eIF4G, part of the cellular translation initiation complex. Targeting this molecule results in decreased production of cellular proteins but does not affect the production of viral proteins, as picornaviruses use internal ribosome entry sites (IRES) for cap-independent translation of mRNA. In this way, the viral protease hijacks the cellular protein production machinery [25,26,27]. The NS3/NS4A protease of the hepatitis C virus cleaves mitochondrial antiviral signaling protein (MAVS) and TIR domain-containing adapter-inducing interferon-β protein (TRIF) to evade the host cell antiviral response [28,29,30].

Various targets of the Zika virus protease were identified, i.e., by mass spectrometry: FAM134B (reticulophagy regulator 1), ATG16L1 (autophagy-related protein 16-1) [31], eIF4G1 (eukaryotic translation initiation factor 4 gamma 1) ([31], as well as our, unpublished results, also identifying other members of the eIF protein family), and Septin-2 [32] are among the most interesting targets. Except for FAM134B, these targets were identified using MS-based methods, using typical proteomic approaches. However, a careful analysis of the reported data showed that none of the protein targets are shared between the published studies. Thus, we explored the reported data in detail by employing classical approaches such as Western blotting, as well as functional approaches based on the activity of particular pathways. Herein, we present an example of such a study in which we failed to confirm protease-mediated or virus-related degradation of the eIF4G1 protein. We were also unable to confirm the beneficial effects of decreasing eIF4G1 on viral replication. Our data stay in contrast to mass spectrometry-based findings, so we believe that extensive discussion of the observed phenomena is necessary to elucidate such inconsistency in the data acquired.

## 2. Results

### 2.1. Expression of Zika Virus Protease in Eukaryotic Cells

**Figure 1:**
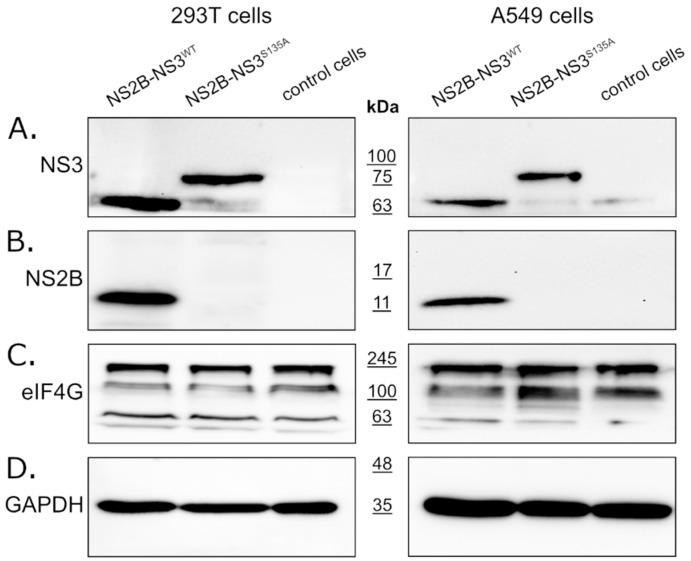
Expression of active and inactive NS2B-NS3 protease from Zika virus in eukaryotic cells does not affect eIF4G1 protein levels. The 293T (left panel) and A549 (right panel) cells expressing NS2B-NS3^WT^ or NS2B-NS3^S135A^ were assessed alongside control cells. Cells were harvested at 48 h after transfection, lysed, and analyzed by Western blotting. Protease expression was confirmed by the presence of NS3 (**A**) and NS2B (**B**) proteins. Anti-eIF4G1 antibody was used to detect and compare changes in eIF4G1 protein abundance in cells expressing active or inactive protease relative to control cells (**C**). The GAPDH protein was used as a reference to ensure that identical amounts of proteins were present in each sample (**D**).

In this study, we expressed full-length NS2B-NS3 protein without linkers. The NS2B-NS3^WT^ protein and its inactive NS2B-NS3^S135A^ variant were expressed from pBudCE4.1 plasmids in 293T (see Figure 1, left panel) and A549 (see Figure 1, right panel) cells. Western blotting with an anti-NS3 antibody (full-length recombinant Zika virus NS3 protein was used as a positive control) detected NS2B-NS3^WT^ and NS2B-NS3^S135A^ in cell lysates prepared from cells collected at 48 h post-transfection. The band corresponding to NS3-NS2B was expected to migrate at ~82 kDa, but the active protease should undergo autocatalytic processing to yield the mature ~68 kDa NS3 protein (Figure 1A). Processing should also result in the generation of the smaller 14 kDa NS2B protein (Figure 1B). Lysates from 293T or A549 cells transfected with empty plasmid were used as controls for each experiment. The samples were prepared in parallel and in the same way as the lysates overexpressing the analyzed proteins. The detection of the expression level of the GAPDH protein was used to confirm that the same amount of proteins was loaded for each sample. All fragments were observed as expected, confirming the activity of the protease, and the protein expression was efficient in both cell lines.

### 2.2. NS3 Protease Does Not Affect eIF4G1 Levels

To verify whether the ZIKV NS3 protease influences eIF4G1 levels, cells expressing either active or inactive protease were analyzed using Western blotting alongside control cells lacking the protease. The results (Figure 1C) showed that eIF4G1 migrated at ~188 kDa, and isoforms with lower molecular masses were also visible. While we observed high variability in eIF4G1 content depending on the culture time, temperature, and general cell conditions (data not shown), there were no differences in protein abundance in cells expressing active or inactive protease or control cells (Figure 1C), and this was the case for both 293T and A549 cells.

### 2.3. Zika Virus Infection Does Not Result in Altered eIF4G1 Levels

**Figure 2:**
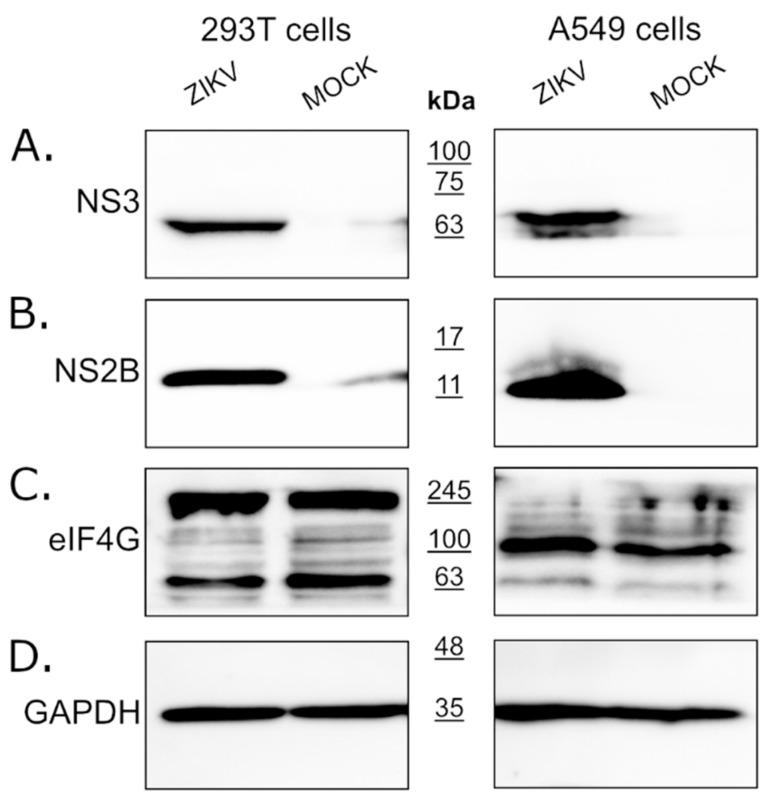
Expression of the eIF4G1 protein in ZIKV-infected and mock-infected cells. The 293T (left panel) and A549 (right panel) cells were infected with ZIKV or inoculated with a mock and analyzed by Western blotting. Virus infection was confirmed by the presence of NS3 (**A**) and NS2B (**B**) proteins. The eIF4G1 protein was detected using an anti-eIF4G1 antibody (**C**). The GAPDH protein was used as a reference to ensure that identical amounts of proteins were present in each sample (**D**).

Since eIF4G1 levels were not affected by the expression of the NS3 protease, we assessed whether the eIF4G1 protein is cleaved or degraded during virus infection using ZIKV infected and mock-infected 293T and A549 cells. Firstly, we confirmed virus replication in cells through NS3 and NS2B protein expression using Western blotting (Figure 2A,B). Levels of the eIF4G1 protein were then assessed in virus-infected and mock-inoculated cells, but there were no significant differences in eIF4G1 protein abundance.

### 2.4. ZIKV Does Not Hamper Production of Cellular Proteins by Altering Levels of Transcription Factors

**Figure 3:**
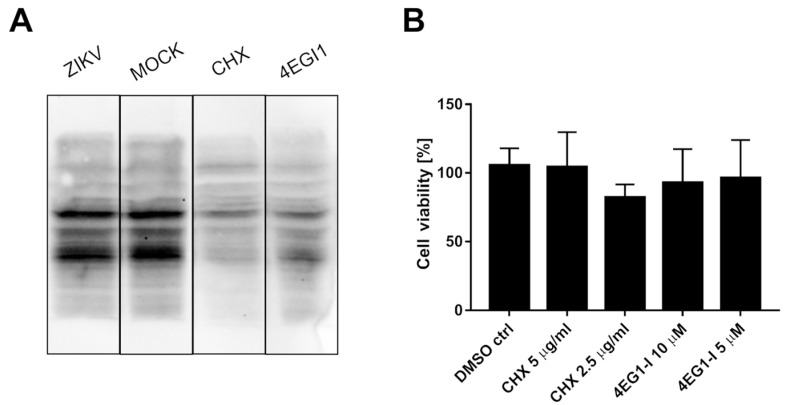
ZIKV infection does not inhibit the translation of host proteins. SUnSet assays were performed on ZIKV- and mock-infected A549 cells and cells treated with cycloheximide or 4EGI1 inhibitors. The presence of puromycin protein was detected using an anti-puromycin antibody (**A**). Cell viability was evaluated relative to control cells treated with DMSO alone. The assay was performed in triplicate, and average values with standard errors are presented (**B**).

It was suggested that ZIKV NS3 cleaves eIF4G1 to redirect the cellular machinery toward viral protein production, which may be independent of cellular transcription factors [31]. To test this hypothesis, the host protein synthesis efficiency was evaluated using surface sensing of translation (SUnSET) assays to measure protein synthesis in cultured cells [33]. Puromycin can mimic the aminoacyl end of aminoacyl-tRNAs and is partially incorporated in polypeptides synthesized during translation. The incorporation rate reflects the rate of mRNA translation. Puromycin incorporation was detected by Western blotting using anti-puromycin antibodies. Two reference inhibitors were also tested: the protein synthesis inhibitor cycloheximide (CHX) and the eIF4G1-specific inhibitor 4EGI1 (Figure 3A). In samples treated with either CHX or 4EGI1, the synthesis of proteins was significantly hampered, but protein synthesis in ZIKV-infected cells was not altered. The cytotoxicity of the inhibitors was also evaluated (Figure 3B).

### 2.5. Overexpression of eIF4G1 Does Not Limit ZIKV Replication

**Figure 4:**
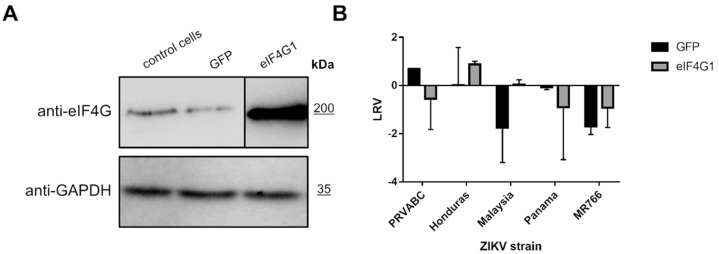
ZIKV replication in eIF4G1-overexpressing cells. (**A**) Western blotting analysis (anti-eIF4G1 antibodies) was performed on 293T cells transfected with plasmid encoding eIF4G1 or GFP. The GAPDH protein was used as a reference to ensure that identical amounts of proteins were present in each sample. (**B**) ZIKV virus replication in 293T cells transfected with plasmid encoding eIF4G1 or GFP. The virus yield was assessed by RT-qPCR. The y-axis represents the log reduction value (LRV) in virus yield in treated samples, and the x-axis corresponds to different ZIKV strains. The assay was performed in triplicate, and average values with standard errors are presented.

To verify whether eIF4G1 expression negatively regulates replication of ZIKV, 293T cells were transfected with a plasmid encoding eIF4G1 (or GFP as a control). Protein levels were verified using Western blotting (Figure 4A), and ZIKV replication was evaluated in non-transfected cells, GFP-expressing cells, and eIF4G-expressing cells. Different strains of the Zika virus were used to ensure that the effect is not limited to a single lineage. Cells were infected, and at a single time point, cell culture supernatants were collected for RNA isolation and subsequent RT-qPCR assessment of the virus yield. Although the virus yields varied depending on the virus strain, no inhibition of virus replication was observed for any of the tested strains. These results show that NS3 mediated loss of function of the eIF4G1 protein was not beneficial for virus replication (Figure 4B).

### 2.6. eIF4G1 Supports Replication of ZIKV

**Figure 5:**
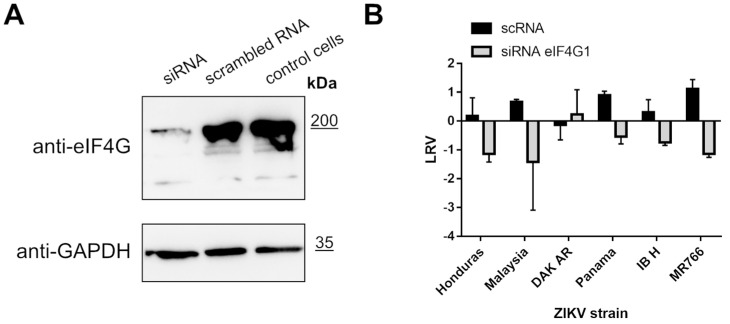
ZIKV replication in cells lacking eIF4G1. A549 cells were transfected with eIF4G1 siRNA or scrambled siRNA. Non-transfected controls were included. The GAPDH protein was used as a reference to ensure that identical amounts of proteins were present in each sample (**A**). ZIKV virus replication in A549 cells transfected with siRNA. The virus yield was assessed by RT-qPCR. The y-axis represents the log reduction value (LRV) in virus yield in treated samples, and the x-axis corresponds to different ZIKV strains. The assay was performed in triplicate, and average values with standard errors are presented (**B**).

To further investigate the role of eIF4G1, we silenced its expression in A549 cells and probed ZIKV replication in these cells. Briefly, cultures were transfected with eIF4G1 siRNA or with scrambled siRNA as a control. Silencing was confirmed by Western blotting using antibodies specific to eIF4G1 (Figure 5A, upper panel). Cells were infected with ZIKV and incubated for 3 days at 37 °C, after which culture supernatants were collected, RNA was isolated and reverse transcribed, and virus replication was evaluated by qPCR. Again, we did not observe an increase in virus production; on the contrary, for some strains, silencing led to inhibition of virus replication relative to control cells or cells transfected with scrambled siRNA (Figure 5B).

## 3. Discussion

This study aimed to verify the involvement of the interaction between ZIKV NS3 protease and endogenous eIF4G1 protein in the remodeling of the cellular microenvironment. Such analysis was a consequence of previous results, based on proteomic data, received by our, as well as other groups, indication involvement of eIF4G1 in viral activity. The NS3 protease is essential for virus replication because it is required for viral protein maturation [34,35,36]. However, viral proteases are generally considered to be highly specific enzymes that co-evolved with the host, and they typically target specific cellular pathways to support viral replication in the host cell or to block recognition of the virus by the host immune system [37,38]. In the case of ZIKV, other possible protease targets were also identified. They are listed in Table 1.

In the present work, we reviewed the published data and verified most of the potential protease targets experimentally by measuring changes in the levels or by observing additional cleaved forms of potential cellular targets in the presence of active or inactive protease. Since, in some cases, the localization or specificity of the protease may differ in the absence of other viral proteins, we also measured the levels of potential NS3 targets in ZIKV-infected cells. Virus-infected cells were also used to verify the biological significance of potential changes in the cellular microenvironment.

We focused on eIF4G1 as a model protease substrate because we believe that the processing of this protein could have straightforward consequences for both host cells and the virus. The eIF4G1 protein is involved in the translation process by serving as a eukaryotic translation initiation factor. Together with eIF4A and eIF4E, eIF4G forms the EIF4F multi-subunit protein complex, which recognizes the mRNA cap and facilitates the recruitment of mRNA to the ribosome. The eIF4G1 serves mainly as a linker that forms a scaffold for the complex [42]. Interestingly, some viruses are reported to target this protein and thereby rewire the cellular machinery and switch off cellular protein production. For example, the coxsackievirus B3 virus-encoded protease cleaves eIF4G1, but the resulting suppression of cellular translation does not affect viral replication since picornaviruses utilize the IRES rather than cap-dependent translation initiation [25,26,27]. Consequently, the complete protein production machinery serves viral replication. Similarly, for some flaviviruses, it was postulated that the 5′-untranslated region (5′-UTR) might act as an IRES, and the NS3 protease encoded in the flaviviral genome may target cellular translation initiation factors [43].

Herein, we first tested whether overexpression of NS2B-NS3 had any effect on levels of the eIF4G1 protein, as reported previously by Hill et al. Notably, the authors of this work performed their analysis using the ZIKV protease expressed in a prokaryotic system, which was purified and mixed with cellular lysates from 293T and A549 cells [31]. In our current work, we expressed part of the ZIKV genome encompassing the NS2B and NS3 proteins. Our approach allowed us to anchor the NS3 protease in the ER membrane via the NS2B cofactor as it occurs in virus-infected cells. Furthermore, using this approach, we were able to monitor whether the protease was active in every experiment because it was autocatalytically (in trans and cis) processing its natural substrate (the NS2B/NS3 junction). We believe that expression of the full-length NS2B-NS3 complex without a linker in the eukaryotic system corresponds better to the natural infection. In our experiment design, both cells transfected with empty plasmid and cells transfected with catalytically inactive mutant were used as negative controls. The protein content analysis did not reveal any significant decrease in eIF4G1 protein levels. However, the experimental setup used in the present study may not be entirely appropriate since it may not accurately mimic protease activity and localization during natural viral infection due to the lack of remaining viral proteins. To ensure that the observed effect was not an artifact, cells were infected with ZIKV, and eIF4G1 levels were measured and compared to mock-inoculated cells, serving as the additional control. Because it remains disputable whether changes in signal transduction reflect a specific decrease in the level of a particular protein, we also tested the effect of ZIKV infection on the production of cellular proteins using the puromycin assay, with appropriate controls that inhibit protein synthesis [44,45]. In cells infected with the Zika virus, we did not observe any changes in host translation, proving that the effect on eIF4G1 potential cleavage is not likely to alter the physiology of the cell. However, modulation may occur locally at the replication site, and while it would improve viral replication, the effect on the whole cell may be too subtle to be detected. For this reason, the effect of eIF4G1 on ZIKV replication was tested by gene silencing and gene overexpression experiments, but the role of the eIF4G1 protein in viral replication remained elusive.

**Figure 6:**
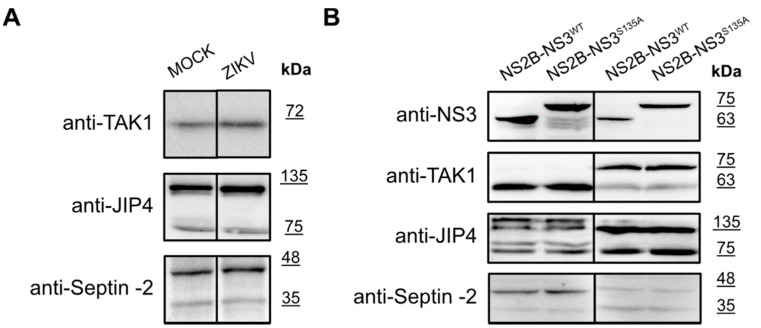
Expression of the TAK1, JIP4, and Septin-2 proteins in ZIKV-infected and mock-infected cells and in cells with expression of active and inactive NS2B-NS3 protease. (**A**) U251 (upper panel), A549 (middle panel), and 293T (bottom panel) cells were infected with ZIKV R103451 Human 2015 Honduras, PRVABC59 or H/PAN/2016 strains, respectively, or inoculated with mock and analyzed by Western blotting. The TAK1, JIP4, and Septin-2 proteins were detected with specific antibodies. (**B**) 293T (left panel) and A549 (right) cells expressing NS2B-NS3^WT^ or NS2B-NS3^S135A^ were analyzed. Cells were harvested at 48 h after transfection, lysed, and lysates were analyzed by Western blotting. Levels and profiles of expression of TAK1, JIP4, and Septin-2 proteins were compared in a cell with an expression of active and inactive. Protease expression was confirmed by the presence of NS3 protein in both cell lines (upper panel).

As listed in Table 1, several proteins were reported as targets for the ZIKV NS3 protease. Intrigued by the results obtained for the eIF4G1 protein, we explored whether autophagy-related protein 16-1 (ATG16L1), c-Jun amino-terminal kinase-interacting protein 4 (JIP4), mitogen-activated protein kinase kinase kinase 7 (TAK1 or MAP3K7), disulfide-isomerase A3 (PDIA3), heterogeneous nuclear ribonucleoprotein A2/B1 (hnRNP A2/B1), aldolase A (ALDOA) [40], ER-localized reticulophagy receptor FAM134B [39], and septin-2 protein [32] may serve as targets for the NS3 protease. For TAK1, JIP4, and Septin-2 proteins, their level or the presence of cleaved forms was verified by a Western blot analysis in infected cells (Figure 6A) as well as in cells with protease expression (Figure 6B).

To our surprise, we could not confirm these previous observations, and we considered why this might be the case. First, in previously cited studies, different expression systems and constructs were employed. In some cases, part of the NS2B cofactor was covalently linked to NS3 by a flexible linker. This is relevant, as it was shown by others that the linker itself might alter the dynamics of the protein and, consequently, the substrate specificity of the protease [46,47]. Second, the soluble version of the protease is not anchored at the membrane, which may also alter the substrate specificity. Third, the localization of the protease in the ER may limit the number of possible targets, and even proteins that may serve as NS3 substrates in biochemical assays may not be cleaved due to the differential spatial distribution.

We are aware of some inconsistency between mass spectrometry-based results, clearly showing changes in the levels of the eIF4G protein family and data presented here. Due to our unpublished results, we were also able to detect changes in this group of proteins (eIF4G1, as well as other translation factors) under the Zika virus influence. We used an N-terminome analysis with the aid of iTRAQ isobaric tags for protein quantitation. Our MS-based results stay, in general, in agreement with the findings of other laboratories presenting their proteomic approaches. However, results indicating the involvement of eIF4G proteins in viral infection were not confirmed by WB- and RT-qPCR-based experiments described in this paper. We were wondering if discrepancies between MS and WB results could be explained simply by the limits of detection (LOD) differences between methods, as there are widely known and easy to compare sensitivity limits described for MS and WB-based experiments. Unfortunately, the problem is not so simple: the LOD-s of both methods strongly depends on the exact assay type. For example, WB sensitivity depends on the membrane used, applied methodology of detection, primary and secondary antibodies overall quality, transfer conditions, and other factors. Depending on the staining, WB LOD varies from hundreds of femtograms (fg) to dozens of picograms (pg) for chemiluminescence or autoradiography detection, to ca. 10–100 pg for colorimetric detection. Examples of detection limit calculations for WB can be found elsewhere [48,49,50,51]. Mass spectrometry is also seen as a very sensitive analytical technique. Typical sensitivity of nanoLC-nanoESI-MS/MS setup with an ion-trap analyzer is estimated as 10 fmol of peptides in a sample (equal to ca. 200–500 pg of protein, depending on its MW), which makes this method, in general, less sensitive than WB. However, more important is the capability of quantitation. For WB, reasonable limits of quantitation (LOQ) should be a single level of magnitude higher than LOD, which still makes this method very sensitive. Working with quantitative MS, the instrument sensitivity depends, i.a., on the MS type: Orbitrap and ICR analyzers are fairly more sensitive than ion-trap or TOF. The recommended quantity of the total protein concentration for a single iTRAQ label is up to 100 µg. Combining four (4-plex) or eight (8-plex) samples in a single assay, we can partially omit the problem of higher LOD achievable than for WB. Moreover, LOD/LOQ comparison between WB and MS leads to the conclusion that the effect barely visible by MS should be clearly visible on WB, contrary to the results presented here. However, in our opinion, the results inconsistency problem lies somewhere else. MS is sensitive enough to detect even minor changes in protein profiles, which, in some cases, could deliver results not relevant for the homeostasis of the intracellular environment. Similar observations were performed for enzymes, which were thought to be involved in opioid addiction/abstinence development. The mass spectrometry detected changes in the levels of some of them, but the results were not translated into the real metabolic changes in the brain, investigated in animal models [52]. The observations led to the conclusion that changes in protein production in the cell do not always reflect homeostatic changes, as some of the proteins can be produced in an inactive form and stored for a long time before activation. It results in the lack of detectable biological change among cell metabolism with simultaneous detection of quantitative changes in protein concentrations by MS. Therefore, the “data inconsistency” problem is not connected to limits of detection or quantitation but is related to data collection method specificity and following interpretation of the acquired results. In the presented case, the problem is even more complex: the mass spectrometry data provide clear evidence showing that eIF4G1, as well as other proteins involved in the translation initiation process, is alternatively regulated after the Zika virus entrance into the cell. It stays in agreement with other viruses’ activities, as described in the introduction, and also seems to be the attractive goal for the virus’s overall activity itself. In contrast, in the experiments, we did not observe any biologically relevant influence of NS2B-NS3 protease on eIF4G1 (and other investigated proteins) and vice versa: the forced up- or down-regulation of eIF4G1 does not have any significant influence on the applied Zika strain’s ability to infect the tested cells. It is worth remembering that we checked whether the obtained results were independent of the cell line (293T, A549, and U251 cells) as well as of the virus strain used (six Zika strains tested). Below we present at least a few explanations of described problem, but unfortunately, at the present stage, they all have speculative value only:

1. Mass spectrometry approaches are sensitive enough to show even minor quantitative changes in proteomic compounds. Depending on the statistical tests applied, the changes in the proteome composition, in general, are not important for cellular homeostasis and can be detected as a potentially significant signal change during purely quantitative measurements. In such cases, the high sensitivity of WB has minor importance. The higher dynamic range of MS analysis (typically 4–5 levels of magnitude or more) could detect small changes in concentrations, usually not detected by WB;
2. MS-based results show significant changes in proteome composition, but due to activation level of down- or up-regulated proteins, the overall effect of detected change for the cell survival/activity is absent or of minor importance. Quantitative MS is unable to distinguish between activated/inactive forms of proteins in the sample. Proteins can be stored in cells in precursor forms, activated by the removal of the N-terminal and signal sequence, or inactivated by ubiquitination. In the proteomic sample, using routine approaches, it is impossible to distinguish between those states of proteins. As a result, we can omit some important changes based on the protein’s current activation state. However, as proteins are usually involved in metabolic pathways, cooperating with others, modifying the receptor’s activities, etc., it is possible to detect changes among results of the primary change. Therefore, during the interpretation of quantitative MS results, we should focus rather on pathways analysis (with the aid of tools such as String-DB) than focus on a single protein. Such an approach could be especially important for the detection of virus/infected cell interactions;
3. MS results could be questionable in extreme cases. If high quantity data, coming from the proteomic analysis, is processed, the identification of the protein bases is usually on a few peptides, which is a common cost of thousands of proteins’ identification in a single assay. It could be not enough for precise determination of exact protein changed in the experiment but valid for the group of the proteins showing a high level of sequence homology. Moreover, MS results could be determined as valid even if they have: (a) relatively low total scores (but still significant), (b) low rankings of the peptides identified, and (c) low quality of some fragmentation spectra included in the data bucket used for protein identification. Thus, search engines set for the data quantitation, to spare calculation time or according to their capabilities, do not take into account simultaneous searches for PTM-s, which, in our case, could significantly influence the final results, omitting quantitative data coming from numerous PTM-modified peptides. Such peptides are simply not recognized during results processing. Quantitative data, coming from iTRAQ labeling or other methods, could also be equivocal enough to give results indicating a change in the whole process (such as metabolic pathway, intracellular process, receptor processing) rather than in the single protein (in our unpublished results, we found at least a few eIF-s with levels changed more or less). Taken together, all these points could seriously influence the MS result for eIF4G1: this protein has 17 identified serine and 4 threonine residues susceptible for phosphorylation, 10 arginine residues with known possible omega-N-methylation, and other known PTM susceptible sites. Significant changes in PTM with a viral infection, even if treated as a side effect, can easily lead to receiving false positives/negatives following significant changes in the quantitative MS analysis. Modified peptides will not be taken into account during data processing, as search engines will be blind towards such PTM if not predicted by the experimenter. It does not mean that such results are misleading or false positive/negative. They simply show what exactly is seen by the MS.

Based on the considerations, especially from point three, our inconsistency in the data is more likely to be related to the MS methodology used. It seems that MS-based findings, which were the inspiration for the presented experiments, show that there is some strong interaction between viral proteins and translation initiation complex rather than direct interaction between the NS2B-NS3 protease and the eIF4G1 translation initiation factor. In conclusion, the elucidation of the observed discrepancies definitely merits further investigation, as problems with data interpretation raised here seem to be important for many types of investigations in life sciences.

In conclusion, our study shows that while the ZIKV NS3 protease is essential for the virus, its biological role in reshaping the cellular microenvironment may be more limited than expected. This may be due to the relatively recent transmission of the virus on a large scale to the human population and incomplete adaptation to the host. Therefore, it would be interesting to analyze virus evolution in humans in the future, including spatial localization and substrate specificity of the NS3 protease.

## 4. Materials and Methods

### 4.1. Cell Culture

The 293T cells (ATCC CRL-3216; human embryonic kidney cells), A549 cells (ATCC CCL-185; lung carcinoma cells), Vero cells (ATCC CCL-81; African green monkey kidney cells), and U251 cells (human glioblastoma cell line) were maintained in Dulbecco’s modified Eagle’s medium (DMEM; Corning, Poland) supplemented with 3% fetal bovine serum (FBS; heat-inactivated; Thermo Scientific, Poland), 100 µg/mL streptomycin, 100 U/mL penicillin, and 5 µg/mL ciprofloxacin (Sigma–Aldrich, Poznan, Poland). The cells were maintained at 37 °C under 5% CO_2_.

### 4.2. Virus Strains: Preparation and Titration

The ZIKV H/PF/2013 strain (from European Virus Archive), ZIKV H/PAN/2016, ZIKV R116265 Human 2016 Mexico, ZIKV Mosquito Mex 2-81, ZIKV PRVABC59, ZIKV MR766, ZIKV IB H 30656, ZIKV FLR, ZIKV R103451 Human 2015 Honduras, ZIKV P 6-740 Malaysia 1966, and ZIKV DAKAR 41,524 strains (all from BEI resources) were employed in this work. The virus stocks were generated by the infection of Vero cells. At 3 days post-infection (p.i.) at 37 °C, the virus-containing medium was collected and titrated. As a control, the mock-infected Vero cells were subjected to the same procedure. The virus and mock aliquots were stored at −80 °C. The virus titration was performed on confluent Vero cells in a 96-well plate according to the method described by Reed–Muench [53]. Briefly, the cells infected with the serially diluted virus were incubated at 37 °C for 3 days, and the occurrence of a cytopathic effect (CPE) was monitored.

### 4.3. Plasmids

The region encoding the NS2B-NS3WT protein was amplified by PCR using a cDNA template generated from the H/FP/2013 Zika virus and appropriate primers (5′ATG CGG TAC CGC CAC CAT GGG CAG CTG GCC CCC TAG CGA A3′; 5′AGC CGG TAC CCT ATC TTT TCC CAG CGG CAA ACT CC3′). The resulting product was digested with NotI-HF and KpnI-HF (New England Biolabs), gel-purified, and cloned into the pBudCE4.1 vector (pBudCE4.1_NS3^WT^). The plasmid encoding the inactive NS2B NS3^S135A^ protease (pBudCE4.1-NS3^S135A^) was obtained using the pBudCE4.1_NS3^WT^ template by employing the QuickChange PCR technique with appropriate primers (5′GGA ACT GCC GGA TCT CCA ATC CTA GAC AAG3′; 5′AGA TCC GGC AGT TCC TGC TGG GTA ATC CAG3′) to change the serine residue at amino acid (aa) position 135 to alanine. The obtained plasmids were verified by DNA sequencing.

### 4.4. Plasmid Transfection

The 293T cells were seeded as described above in 6- or 24-wells plates (TPP, Switzerland) and cultured for 24 h. When 60% confluency was reached, cells were transfected using polyethyleneimine (PEI; Sigma–Aldrich, Poland). For transfection in 6-wells plates, 4 μg plasmid DNA was mixed with 250 μL Opti-MEM medium (Thermo Scientific) and 4 μg PEI. For transfection in 24-well plates, 1 μg/well plasmid DNA was mixed with 100 μL Opti-MEM medium with 1 μg PEI. After a 30 min incubation at room temperature, the mixture was added dropwise onto cells. Four hours later, the supernatant was discarded, fresh medium was added, and cells were further incubated at 37 °C.

The A549 cells were seeded in 6-wells plates and cultured for 24 h. When 80% confluency was reached, cells were transfected with Lipofectamine 2000 (Thermo Scientific) according to the manufacturer’s protocol. Briefly, 2.5 μg plasmid DNA was mixed with 300 μL Opti-MEM medium with 5 μL Lipofectamine 2000. After a 5 min incubation at room temperature, the mixture was added dropwise onto cells. Four hours later, the supernatant was discarded, fresh medium was added, and cells were further incubated at 37 °C.

For the expression of active and inactive virus protease, pBudCE4.1-NS3^WT^ or pBudCE4.1-NS3^S135A^, plasmids were employed, respectively. For the eIF4G1 overexpression, cells were transfected with pcDNA3 HA eIF4GI plasmid or control green fluorescent protein (GFP)-expressing plasmid (pMAX-GFP plasmid, Lonza). The pcDNA3 HA eIF4GI (1-1599) was a gift from Nahum Sonenberg (Addgene plasmid #45640; http://n2t.net/addgene:45640; RRID Addgene_45640, accessed date: January 2019). The efficiency of expression was verified by Western blotting.

### 4.5. siRNA Transfection

For small interfering RNA (siRNA) transfection, A549 cells were seeded in 24-wells plates, and siRNA was transfected once the confluency reached 80% using RNAiMAX Lipofectamine (Thermo Scientific), according to the manufacturer’s protocol. Next, 5 pmol eIF4G1 siRNA (Sigma–Aldrich, St. Louis, MO, USA; Cat. No EHU066831) or control scrambled RNA (Santa-Cruz Biotechnology; Cat. No sc-44237) was mixed in 125 μL Opti-MEM medium containing 3.5 μL transfection reagent. After a 5 min incubation at room temperature, the mixture was added to cells dropwise. The efficiency of eIF4G1 silencing was verified at 24–72 h post-transfection using Western blotting.

### 4.6. Virus Infection

The 293T cells, A549 cells, and U251 cells were seeded in 6-wells plates and cultured at 37 °C. When 90–100% confluency was reached, cells were inoculated with ZIKV at 2000 TCID 50/mL for 293T cells or 400 TCID 50/mL for U251 and A549 cells. The mock cultures were inoculated with an identical volume of mock samples. All cultures were incubated for 2 h at 37 °C under 5% CO_2_ in DMEM with 2% FBS, 100 µg/mL streptomycin, and 100 IU/mL penicillin. After incubation, cells were washed twice with phosphate-buffered saline (PBS) and incubated as described above. At 3 days p.i., culture supernatants were collected, viral RNA was isolated, and the yield was quantified by reverse transcription-quantitative PCR (RT-qPCR). Cells were collected for the Western blotting analysis.

### 4.7. SUnSET-Puromycin Assay

A549 and U251 cells were seeded in 12-well plates and cultured for 48 h. When 80% confluency was reached, cells were inoculated with the Mexico ZIKV strain at 400 TCID 50/mL (or an identical volume of the mock culture). Alternatively, 10 μg/mL or 5 μg/mL of the translation inhibitor cycloheximide (CHX; stock solution 100 mg/mL; Sigma–Aldrich) was added to A549 cells and U251 cells, respectively, or 10 μM eIF4G1 inhibitor (4EGI-1; stock solution 10 mM; Biotechne) [44] was added. At 48 h p.i., cells were washed twice with PBS and incubated in unsupplemented DMEM for 2 h at 37 °C. The supernatant was discarded, fresh DMEM medium supplemented with 3% FBS, and 1 μM puromycin (stock solution; Merck, Rahway, NJ, USA) was added, and cells were further incubated for 30 min at 37 °C. Subsequently, cells were collected for Western blotting analysis.

### 4.8. SDS-PAGE and Western Blotting

Cells grown in 6-well, 12-well, or 24-well plates were lysed for 30 min on ice in 200 µL, 100 μL, or 50 μL RIPA buffer (50 mM TRIS, 150 mM NaCl, 1% NP-40, 0.5% sodium deoxycholate, 0.1% SDS, pH 7.5), respectively. Next, samples were centrifuged (10 min at 13,000× *g*, 4 °C), and the pelleted cell debris was discarded. The total protein concentration in each sample supernatant was quantified using the bicinchoninic acid (BCA) method (Pierce BCA Protein Assay Kit; Thermo Scientific) according to the manufacturer’s protocol. Supernatants were mixed 5:1 with a denaturing buffer (202.5 mM TRIS pH 6.8, 10% SDS, 15% β mercaptoethanol, 30% glycerol, 0.3% Bromophenol Blue) and boiled at 95 °C for 5 min.

For the detection of proteins, 30 μg of protein lysates were loaded, and separated SDS-PAGE (12% gel) over 2 h at 120 V. BlueStar Plus Prestained Protein Markers (NIPPON Genetics, Germany) were used for reference. Subsequently, gels were subjected to wet electrotransfer onto methanol-activated polyvinylidene difluoride membrane (PVDF; GE Healthcare, Poland) in 25 mM TRIS, 192 mM glycine, 20% methanol buffer for 1 h at 100 V. Then, the membrane was blocked with 5% skimmed milk (BioShop, Burlington, Canada) in TRIS-buffered saline (20 mM TRIS, 0.5 M NaCl, pH 7.5) supplemented with 0.05% Tween 20 (TBS-T) by overnight incubation at 4 °C. To detect NS3 protein, the membrane was incubated with a rabbit anti-NS3 antibody (1:1000; GeneTex, Irvine, CA, USA) followed by a secondary goat anti-rabbit antibody (1:20,000; Dako, Glostrup, Denmark) conjugated with horseradish peroxidase (HRP). To detect the eIF4G1 protein, membranes were incubated with a rabbit anti-eIF4G1 antibody (1:1000; Thermo Scientific) followed by a secondary goat anti-rabbit antibody (1:20,000; Dako) conjugated with HRP. For puromycin detection, membranes were incubated with mouse anti-puromycin antibody (1:10,000; Merck) followed by a secondary rabbit anti-mouse antibody (1:20,000; Dako) conjugated with HRP. To detect the GAPDH protein, membranes were incubated with a rabbit anti-GAPDH antibody (1:5000; Cell Signaling) followed by a secondary goat anti-rabbit antibody (1:20,000; Dako) conjugated with HRP. Additionally, for TAK1, JIP4, and Septin-2 detection, membranes were incubated with rabbit anti-TAK1 antibody (1.5 μg/mL; Thermo Scientific), mouse anti-JIP4 antibody (1:500; Santa Cruz Biotechnology, Dallas, TX, USA), and mouse anti-Septin-2 antibody (1:2000; Santa Cruz Biotechnology), respectively. As a secondary, a rabbit anti-mouse antibody (1:20,000; Dako) or goat anti-rabbit antibody (1:20,000; Dako) conjugated with HRP. All antibodies were diluted in 1.5% skimmed milk in TBS-T. The signal was developed using Immobilon Western Chemiluminescent HRP Substrate (Millipore, Warsaw, Poland) and recorded with a ChemiDoc Imaging System (Bio-Rad, Warsaw, Poland).

### 4.9. Isolation of Nucleic Acid and Reverse Transcription

Viral RNA was isolated from 100 µL cell culture supernatant using a viral DNA/RNA Isolation Kit (A&A Biotechnology, Gdansk, Poland) according to the manufacturer’s protocol. Reverse transcription was carried out using a High Capacity cDNA Reverse Transcription Kit (Thermo Scientific) according to the manufacturer’s protocol. cDNA samples were prepared in 10 µL volumes using a High Capacity cDNA Reverse Transcription Kit (Thermo Scientific) according to the manufacturer’s instructions. The reaction was carried out for 10 min at 25 °C, 120 min at 37 °C, and 5 min at 85 °C.

### 4.10. Quantitative PCR (qPCR)

The Zika virus RNA yields were assessed using real-time PCR on a 7500 Fast Real-Time PCR instrument (Thermo Scientific, Poland). ZIKV cDNA was amplified in a reaction mixture containing 1× TaqMan Universal PCR Master Mix (RT-PCR mix; A&A) in the presence of FAM/TAMRA (6-carboxyfluorescein/6-carboxytetramethylrhodamine) probe (5’CGG CAT ACA GCA TCA GGT GCA TAG GAG3’; 100 nM) and primers (5’TTG GTC ATG ATA CTG CTG ATT GC3’ and 5’CCT TCC ACA AAG TCC CTA TTG C3’; 450 nM each). The reaction was carried out for 2 min at 50 °C and 10 min at 92 °C, followed by 40 cycles at 92 °C for 15 s and 60 °C for 1 min. DNA standards were subjected to qPCR along with the cDNA. Rox was used as a reference dye.

## Figures and Tables

**Table 1:** Reported NS3 protease targets in the cell. Abbreviations: MS—mass spectrometry, WB—Western blotting.

Protein	Function	Protease Expression	Cellular Model/ Identification	Ref.
ATG16L1	Autophagy	Expression in the prokaryotic system; construct based on 48–100 aa residues of NS2B and 1–178 aa of NS3 (protease domain)	protease substrate verification in the cellular lysate of 293T and A549 cells treated with purified protease/MS and WB	[31]
eIF4G1	Translation	Expression in prokaryotic system; construct based on 48–100 aa residues of NS2B and 1–178 aa of NS3 (protease domain)	protease substrate verification in cellular lysate of 293T and A549 cells treated with purified protease/MS and WB	[31]
FAM134B	Reticulophagy	Expression in eukaryotic cells; the NS2B-NS3 protein-coding region was amplified from cDNA produced from HBMEC infected with ZIKV MR766; cells transfection	HBMEC, U2OS, 293T and HeLa cells/WB and fluorescence microscopy	[39]
Septin-2	Cytokinesis	Expression in eukaryotic cells, cells transfection, and transduction with lentiviral vectors	HeLa and 293T cells and human neural progenitor cells/MS, WB, fluorescence microscopy, pull-down assay	[32]
Disulfide isomerase A3 (PDIA3)	ER stress response	Expression in eukaryotic cells, cells transfection; construct based on 48–94 aa residues of NS2B and 1–188 aa of NS3 (protease domain)	293T and A549 cells/MS and WB	[40]
Aldolase A (ALDOA)	Glycolysis	Expression in eukaryotic cells, cells transfection; construct based on 48–94 aa residues of NS2B and 1–188 aa of NS3 (protease domain)	293T and A549 cells/MS and WB	[40]
Nup98, Nup153, and TPR	Formation of nuclear pore complex	Expression in eukaryotic cells, cells transfection	Huh-7 cells/MS, WB, and fluorescence microscopy	[41]

## Data Availability

Most of the data used during the preparation of the manuscript are included in the Results and Discussion sections. However, for any additional details of the procedures and the results’ original raw files, please contact the corresponding authors.
